# Emotional dysmetria after cerebellar-pontine stroke: a case report

**DOI:** 10.1186/s13256-023-04294-1

**Published:** 2023-12-15

**Authors:** Rebecca M. Long, Michèle DuVal, Bridget Mulvany-Robbins, Amanda N. Wagner, Glen C. Jickling

**Affiliations:** 1 Cumming School of Medicine, University of Calgary, Calgary, Canada; 2 Neuroscience and Mental Health Institute, Faculty of Medicine and Dentistry, University of Alberta, Edmonton, Canada; 3 Department of Medicine, Division of Neurology, University of Alberta, Edmonton, Canada

**Keywords:** Pseudobulbar affect, Ischemic stroke, Emotional lability, Depression, Cerebellum, Case report

## Abstract

**Introduction:**

Pseudobulbar affect, or emotional dysregulation, commonly occurs following stroke. However, it is frequently missed in cases involving the cerebellum, resulting in a lack of treatment, which can directly impact stroke rehabilitation.

**Case presentation:**

A 63-year-old Caucasian female with no history of mood disorders presented with gait instability, dysarthria, and right sided hemiplegia, secondary to cerebellar and pontine ischemic stroke from a basilar occlusion. She underwent endovascular therapy and her deficits gradually improved. However during recovery she began to develop uncontrollable tearfulness while retaining insight that her emotional expression was contextually inappropriate. She was treated with a selective serotonin reuptake inhibitor with reported improvements in her emotional regulation at one year follow up.

**Conclusion:**

This case highlights cerebellar injury as a potential cause of poorly regulated emotions, or an emotional dysmetria. The recognition of this disorder in patients with cerebellar or pontine strokes is critical, as untreated pseudobulbar affect can impact future stroke rehabilitation.

## Background

Pseudobulbar affect (PBA) is a dysregulation of emotions that can occur in 17–20% of patients with acute ischemic stroke [1]. Typical symptoms include crying or laughter that is disproportionate and/or inappropriate to context, not under full voluntary control, and can occur multiple times a day [2]. Between emotional outbursts, symptoms generally resolve. Unfortunately, PBA is frequently underrecognized and thus physicians may miss opportunities to treat and improve care in patients following stroke [3]. Early recognition and treatment of PBA can have significant implications on quality of life and patient engagement with rehabilitation, resulting in improved recovery post stroke.

While PBA is commonly associated with damage to the cortex and limbic system, it’s important to emphasize that it can also be a concern in patients with cerebellar and pontine strokes. Emotion is distributed widely across the brain involving cortical and subcortical structures, including the limbic system, brainstem and cerebellum [4]. PBA can result from injury or dysfunction anywhere along the cortico-ponto-cerebellar pathway [5]. Other disorders of emotional regulation, including depression and mania, have also been described in the context of cerebellar lesions, as recently reviewed by Frazier *et al.* [6], and is also known to occur as cerebellar cognitive-affective syndrome in children following surgical removal of posterior fossa tumours [7]. Within the cerebellum, the hemispheres are the main regions that contribute to PBA. The cerebellar hemispheres contain numerous tracts that project, via the pons, to and from the dorsolateral and dorsomedial prefrontal cortex, posterior parietal region, cingulate gyrus, and to limbic structures including the septal nuclei and the hippocampus [8]. The perception and experience of emotion involves this complex network for not only appropriate expression of emotions, but also the detection of emotionally charged stimuli, and assessment of the social context. The cerebellum specifically plays a role in the coordination and control of emotion expression in appropriate social circumstances, and if this coordination is impaired, a poorly measured emotional response, or a dysmetria of emotions, can occur. Emotional and contextual information from the cerebrum is sent to the cerebellum via cortico-ponto-cerebellar connections, and coordinates with the effectors in motor cortex, brainstem, and cranial nerve nuclei to express the perceived emotion [9]. If this pathway is disrupted, it may result in a discoordination of outward emotional expression, or PBA [10].

## Case presentation

**Fig. 1:**
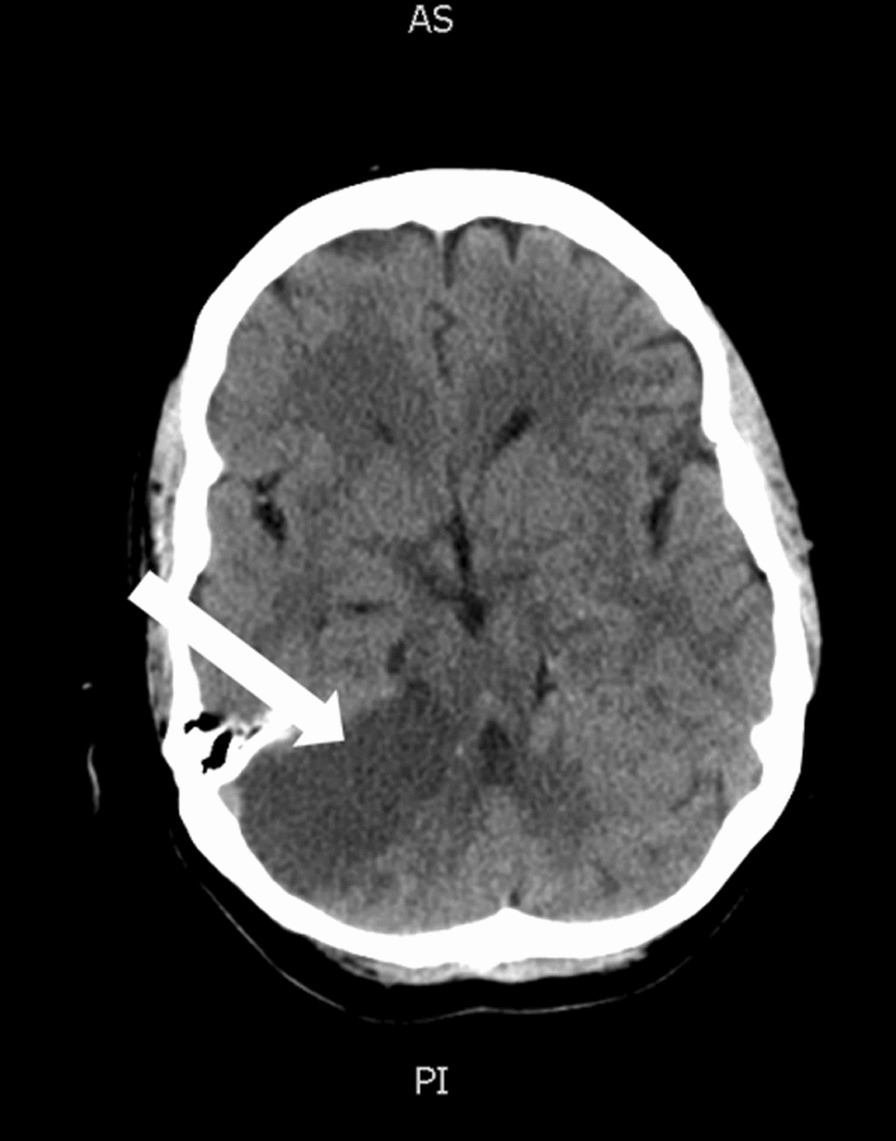
Plain computed tomography brain scan showing ischemia of the lateral right cerebellar hemisphere (arrow)

**Fig. 2:**
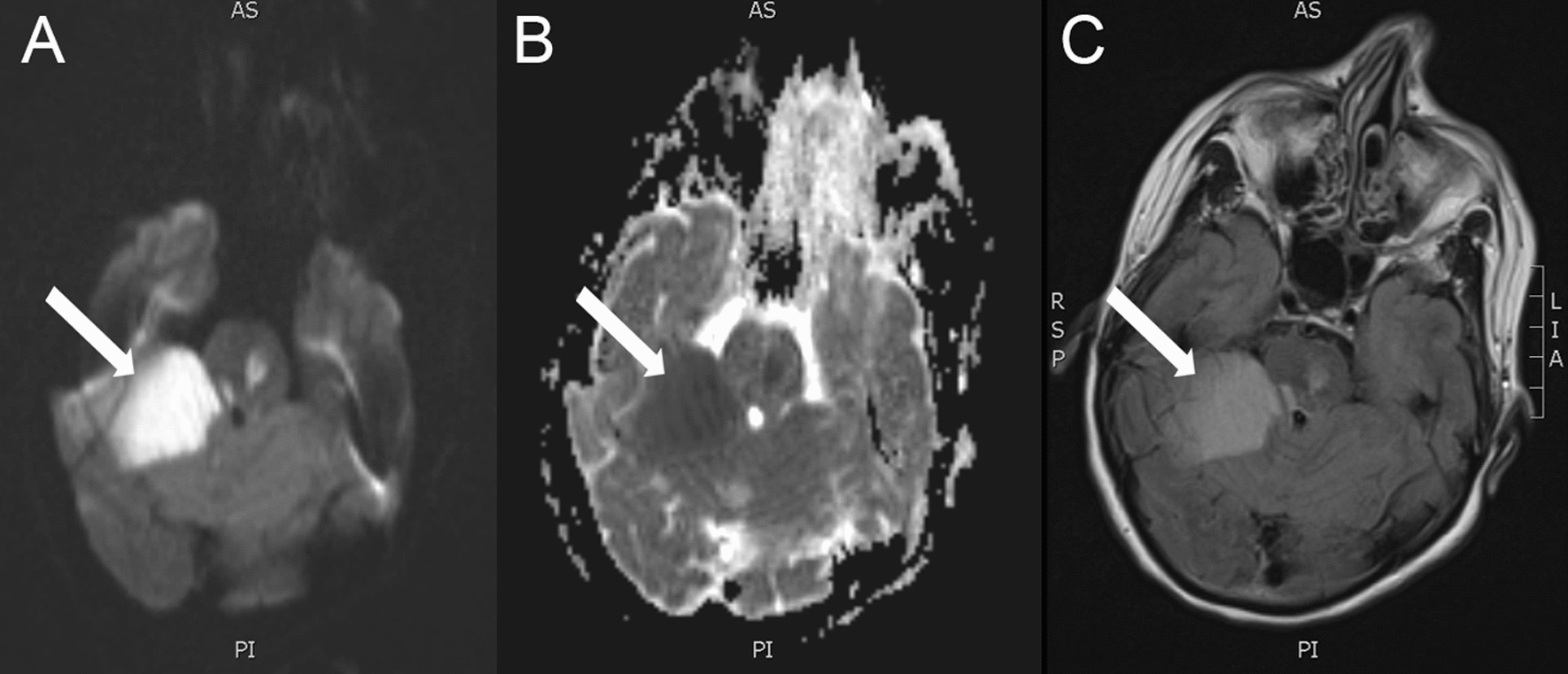
MR Brain with DWI (**A**), ADC (**B**), and FLAIR (**C**) sequences. The white arrows demonstrate a large territory of restricted diffusion in the right cerebellar hemisphere, with an additional focus in the left pons

A 63-year-old Caucasian female with diabetes and dyslipidemia presented with sudden onset gait instability, right-sided hemiplegia, and severe dysarthria. She was previously independent, required no additional support and lived in a private home. The symptoms started 1.5 h prior to her presentation. Her NIHSS was 13. Neurovascular imaging revealed a proximal occlusion of her basilar artery, which was successfully recanalized with endovascular therapy five hours after her symptom onset. She regained antigravity strength on the right side, with subsequent brain imaging showing bilateral cerebellar and pontine ischemia (Figs. 1, 2), which was the likely cause of her deficits.

During her recovery in hospital, she began to experience sudden outbursts of tearfulness in response to routine questions. The tearfulness was often so intrusive, it prevented her from conversing. Additionally, bedside PHQ-9 was completed 7 days after her stroke with a score of 18, suggesting moderately severe depressive symptoms. A diagnosis of PBA was made due to her uncontrollable tearfulness. Interestingly, she was able to recognize that her emotional response was inappropriate given the current context, a feature that is not always present in PBA. She denied any history of depression, anxiety, or other mood disorders. The emotional lability limited her capacity to communicate effectively with clinical staff and engage in rehabilitation as she was unable to control her tearfulness during interactions, which resulted in increased social stress, exacerbating the situation further. She was started on escitalopram 10 mg and noted an improvement in emotional lability over the ensuing weeks, allowing better engagement in her post-stroke rehabilitation.

She was transitioned to a long-term rehabilitation centre for a few months where she continued to improve. At her follow-up visits 1 year later, she still had some residual right sided hemiparesis and right arm and leg ataxia, but her mood and emotion regulation have remained stabilized on escitalopram. She remains fully independent for her basic ADLs and requires minimal assistance with some instrumental ADLs.

## Discussion

Emotion processing occurs across cortical, subcortical, and cerebellar regions within the brain [4–9], and damage along this pathway can result in varying degrees of emotional impairment. In patients with PBA, their insight into their emotional response may also be affected. While literature and formal investigation is lacking, this impairment of insight can potentially aid in the localization of the lesion along the cortico-ponto-cerebellar pathway, with cortical lesions anecdotally resulting in loss of emotional insight, and lesions of the pons or cerebellum reportingly sparing insight [11]. This dissociation follows the idea that the cortical and subcortical regions of the brain are more involved in emotional processing and interpretation, while the pons and cerebellum play a role in modulating the motor output. Currently, this relationship has not been fully established, and represents an opportunity for further research. While not supported in the current literature, we speculate that intact cortical and subcortical regions may be required for retained insight, as shown in the current presentation. In a patient with a cerebellar/pontine stroke, the presence of increased emotionality with retained insight may provide clues that uniquely link cortical and subcortical areas with emotional reasoning in the context of PBA. In addition to impacting a person’s interpretation of their resulting emotional state, untreated PBA can have a deleterious impact on post-stroke recovery in other ways, making recognition of PBA in cerebellar stroke critical. The dysmetric expression of emotion often causes patients distress and embarrassment, leading to curtailment of social engagement, and therefore limited participation in rehabilitation and interaction with others. As such, prompt recognition and initiation of treatment can be advantageous in expediting effective rehabilitation practices. Additionally, PBA can exacerbate pre-existing anxiety and depression, further hindering rehabilitation efforts [10]. While there are no reports on how PBA directly affects the outcome of stroke rehabilitation, it is known that emotional impairment such as depression can complicate and delay stroke rehabilitation [12]. Further, caregivers report they endure more distress and upsetting experiences than caregivers of non-PBA patients [10]. Overall, early treatment of PBA can reduce emotional outbursts [13, 14] and may help improve stroke outcomes. Antidepressants and dextromethorphan-quinidine are commonly used in the treatment of PBA, with adjunctive cognitive therapy also being of potential benefit [15].

## Conclusion

In conclusion, while PBA can occur in patients with damage to the cortex and limbic system, it can also occur with ischemic stroke of the cerebellum and pons. Here, we suggest that PBA should be considered when emotional dysmetria is identified. Early recognition is important, as prompt treatment can improve quality of life, reduce associated depression and anxiety, and increase engagement in post-stroke rehabilitation. The presented case highlights the role of ischemic injury to the cerebellum and pons as a cause of PBA with retained insight into emotional response. Recognition of PBA in the presented patient permitted early initiation of a selective serotonin reuptake inhibitor, which reduced her emotional lability and aided her participation in post-stroke rehabilitation.

## Data Availability

Not applicable.
